# Establishment of a diabetes mellitus type 1 model in the common marmoset

**DOI:** 10.1038/s41598-019-51199-5

**Published:** 2019-10-10

**Authors:** Wenji Yuan, Satsuki Fukuda, Takashi Inoue, Hitoshi Okochi, Erika Sasaki, Masayuki Shimoda

**Affiliations:** 1 Department of Pancreatic Islet Cell Transplantation, National Center for Global Health and Medicine, 1-21-1 Toyama, Shinjuku-ku, Tokyo, 162-8655 Japan; 2 Department of Regenerative Medicine, National Center for Global Health and Medicine, 1-21-1 Toyama, Shinjuku-ku, Tokyo, 162-8655 Japan; 3 Department of Marmoset Biology and Medicine, Central Institute for Experimental Animals, 3 Chome-25-12 Tonomachi, Kawasaki-ku, Kawasaki-shi, Kanagawa-ken 210-0821 Japan

**Keywords:** Type 1 diabetes, Animal disease models

## Abstract

Common marmosets have attracted considerable attention as a small standard primate model in biomedical research. However, no marmoset diabetes model is available. Here, we established a marmoset diabetes model via the combination of partial pancreatectomy and intravenous streptozotocin (STZ). A partial pancreatectomy was performed in 11 common marmosets and multiple STZ doses were intravenously administered. Diabetes was diagnosed upon sustained hyperglycaemia (nonfasting blood glucose level >200 mg/dl). Blood glucose and biochemistry were periodically assessed, in addition to glucose tolerance testing, continual blood glucose determination using a continuous glucose monitoring system, urine testing and histological evaluation. In 8 of the 11 animals (73%), diabetes mellitus was induced. The diabetic marmosets also showed abnormal intravenous and oral glucose tolerance test results. Blood glucose levels decreased in response to human insulin administration. The hyperglycaemic state was irreversible and persisted for more than 3 months, and the animals’ condition was manageable via daily insulin administration. Thus, diabetes can be successfully induced and maintained in the common marmoset via partial pancreatectomy and STZ administration. This protocol effectively generates a valuable animal model for studying disease pathogenesis, risk factors and therapeutic interventions, including islet transplantation.

## Introduction

Type 1 diabetes mellitus, characterised by progressive destruction of insulin-producing cells, is an organ-specific autoimmune disease. For patients with brittle type 1 diabetes mellitus, islet transplantation is an effective and safe treatment^1,2^. However, there are several limitations to this approach. One of the most serious drawbacks is the severe shortage of donors^3^. Thus, a promising alternative due to its potentially unlimited supply involves transplantation of pancreatic islet-like cells produced from embryonic stem cells and somatic stem cells^4,5^. Although these cell therapies are highly encouraging approaches, the preclinical effectiveness and safety data need to justify the initiation of any clinical trials. Based on the close phylogenetic relationship of nonhuman primates (NHPs) with humans and their metabolic and hormonal systems, NHPs are excellent models for preclinical diabetes research^6–10^. Several NHPs, such as rhesus monkeys, cynomolgus monkeys and baboons, have been used as transplantation models in clinical studies.

Nonetheless, alternative NHP models are desired. The common marmoset is a useful animal model in fields such as neuroscience and stem cell research because of their compact size and biological characteristics^9,10^. The common marmoset is also expected to be suitable for islet transplantation research. However, a marmoset diabetes model has not yet been established.

Pancreatectomy or streptozotocin (STZ) can be used to induce a diabetes mellitus type 1 model in many animal species^11,12^. STZ, transported into beta cells by the glucose transporter-2 receptor, induces beta cell death. However, particularly at high doses, STZ administration can cause nephrotoxicity and hepatotoxicity. In addition, STZ is less effective for beta cell destruction in the marmoset^13^. Indeed, our preliminary work showed that STZ administration alone failed to induce diabetes in the marmoset. High-dose STZ administration did not cause diabetes but led to liver and renal failure (data not shown).

On the other hand, total pancreatectomy is a difficult procedure in the marmoset due to tight adherence of the pancreas to the intestine and other anatomical reasons. Partial pancreatectomy is a simpler and safer technique, but there is potential for continued functioning or regeneration of the remaining beta cells and the animal may not develop diabetes.

Accordingly, in this study, we investigated whether the combination of partial pancreatectomy and STZ administration could successfully and safely induce diabetes mellitus type 1 in the common marmoset and physiologically and histologically assessed the model.

## Results

### Blood glucose management

After partial pancreatectomy, nonfasting blood glucose values ranged from about 100 to 140 mg/dl, without a significant increase from baseline. About 20 to 30 days after the partial pancreatectomy, the marmosets received the first STZ injection (160 mg/kg). If the first STZ administration did not affect blood glucose levels, additional STZ was administered 1 to 4 additional times at intervals of 1 to 2 months (Table 1). The additional STZ dose was adjusted to the condition of the marmoset (100 to 160 mg/kg).

**Table 1:** Number of STZ administrations to each animal to induce hyperglycaemia.

Individual number	Sex	Initial dose of STZ	Second dose of STZ	Third dose of STZ	Fourth dose of STZ	Fifth dose of STZ	Outcome
1	Female	160 mg/kg	160 mg/kg	120 mg/kg	100 mg/kg		Diabetic
2	Female	160 mg/kg	160 mg/kg	100 mg/kg	100 mg/kg	140 mg/kg	Dead
3	Female	160 mg/kg	160 mg/kg	100 mg/kg	120 mg/kg	160 mg/kg	Diabetic
4	Male	160 mg/kg	100 mg/kg	140 mg/kg			Diabetic
5	Female	160 mg/kg	140 mg/kg	100 mg/kg	120 mg/kg	140 mg/kg	Normoglycaemia
6	Male	160 mg/kg	160 mg/kg	160 mg/kg	160 mg/kg		Dead
7	Male	160 mg/kg	160 mg/kg				Diabetic
8	Male	160 mg/kg	160 mg/kg				Diabetic
9	Male	160 mg/kg	160 mg/kg				Diabetic
10	Male	160 mg/kg	160 mg/kg	140 mg/kg			Diabetic
11	Male	160 mg/kg	160 mg/kg				Diabetic

After the initial STZ injection, 100–160 mg/kg STZ was administered over between 1 and 4 additional injections for a total of 2–5 injections.

**Figure 1:**
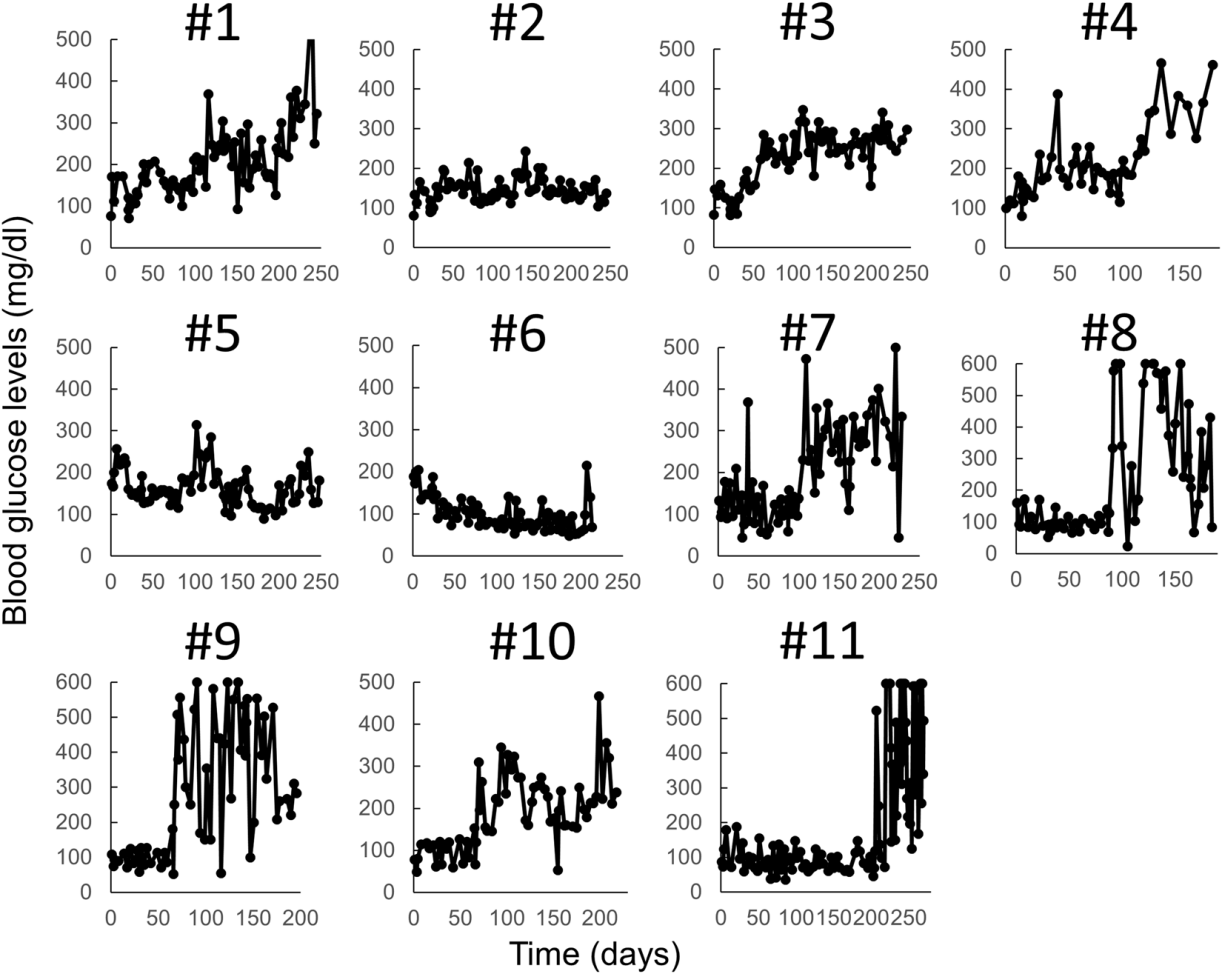
Nonfasting blood glucose level changes of individual marmosets after partial pancreatectomy and administration of STZ. The horizontal axis represents the number of days after partial pancreatectomy. The vertical axis represents the blood glucose level (mg/dl). Eight marmosets (#1, #3, #4, #7, #8, #9, #10, #11) became diabetic, whereas 3 marmosets (#2, #5, #6) remained normoglycaemic. Marmosets #2 and #6 died after multiple STZ injections, probably due to liver failure. Note: the indicated blood glucose levels were sometimes lower than the actual levels because hyperglycaemic marmosets were maintained by insulin injection.

Of the 11 animals, 8 showed elevated nonfasting blood glucose levels that exceeded 200 mg/dl (Fig. 1). In addition, 6 of the 7 males (86%) and 2 of the 4 females (50%) developed diabetes, with no significant difference between the sexes. There were no differences between the two groups in terms of preoperative body weight or blood tests, including renal function (Supplemental Figs S1 and S2). The nonfasting blood glucose levels were maintained at >200 mg/dl for more than 3 months. Body weight was often reduced by the pancreatectomy, STZ administration and diabetic conditions (Supplemental Fig. S2). During the observation period, 6 diabetic marmosets that showed severe clinical signs such as marked weight loss (>20%), hyposthenia, dehydration, anorexia and cutaneous abscess were administered exogenous human insulin to manage their condition. Invasive tests were not performed in some marmosets because their general condition had deteriorated at the time of be examination and could not be improved by insulin administration and other intensive care.

### Intravenous glucose tolerance tests and oral glucose tolerance tests

**Figure 2:**
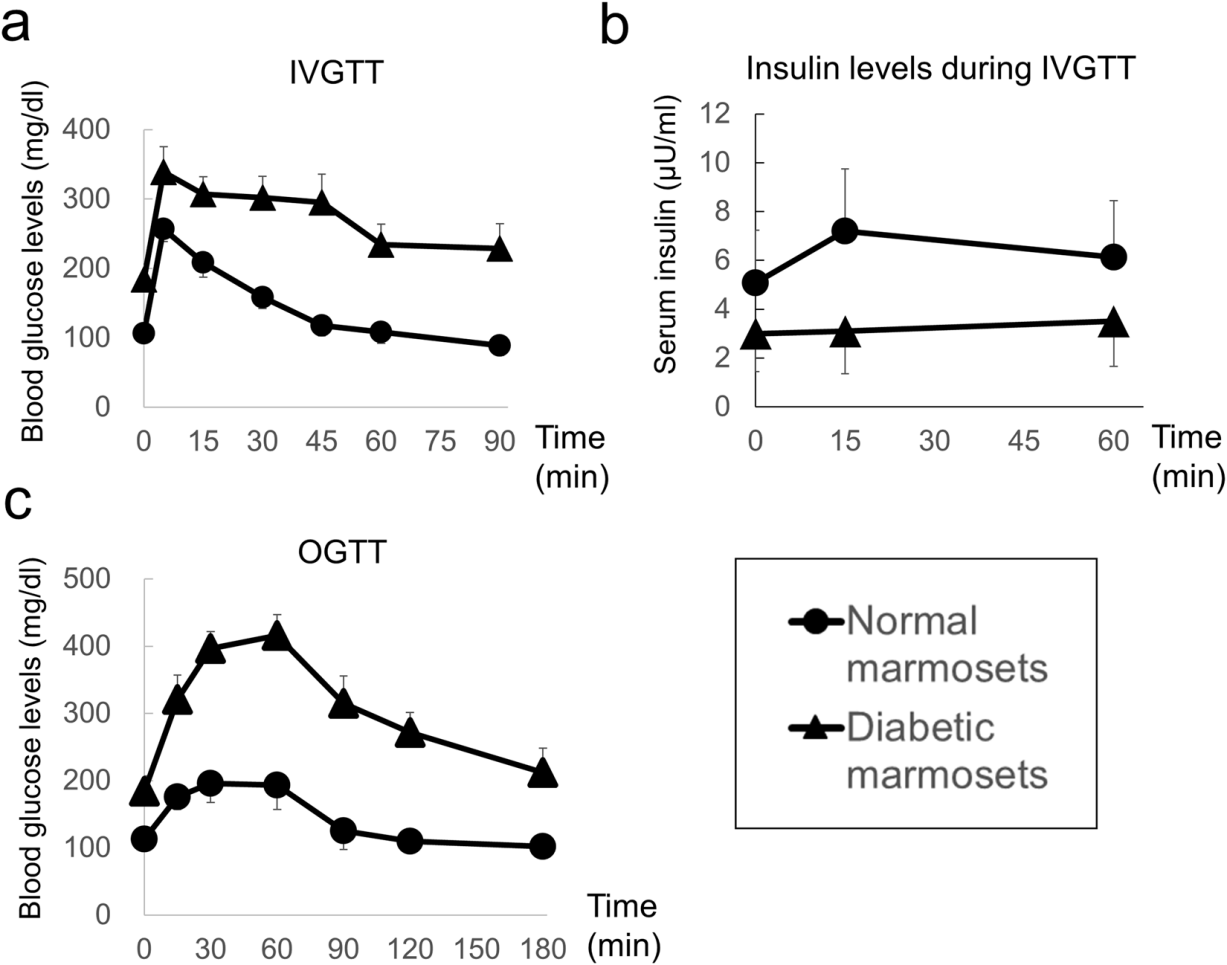
IVGTT and OGTT in normal and diabetic marmosets. (**a**) Blood glucose levels during IVGTTs. Black circles: normal marmosets (N = 9). Black triangles: diabetic marmosets (N = 7). (**b**) Serum insulin levels 0, 15 and 60 min after glucose challenge. Black circles: normal marmosets (N = 6). Black triangles: diabetic marmosets (N = 4). (**c**) OGTT in normal and diabetic marmosets. Black circles: normal marmosets (N = 9). Black triangles: diabetic marmosets (N = 6). Values are the mean ± standard error. Note: some marmosets were not investigated due to their poor condition.

After an overnight fast (16 h), glucose solution (0.5 g/kg) was injected through the tail vein for intravenous glucose tolerance tests. Diabetic marmosets showed abnormal glucose tolerance (Fig. 2a). The insulin levels of diabetic marmosets were lower than those of normal marmosets and did not respond to the glucose challenge (P < 0.05; Fig. 2b).

After an overnight fast (16 h), 2 g/kg glucose solution was orally administered for oral glucose tolerance tests (OGTTs) (Fig. 2c). During the OGTT, the animals in the diabetic marmoset group showed significantly higher peak glucose levels than normal marmosets (P < 0.05). After 120 min, the blood glucose level in the diabetic marmosets exceeded 200 mg/dl, indicating impaired glucose clearance.

### Insulin tolerance test

**Figure 3:**
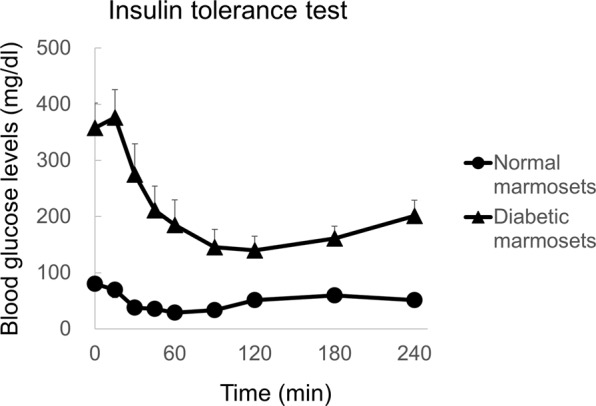
Insulin tolerance test. Blood glucose levels in normal and diabetic marmosets after human insulin administration. Black circles: normal marmosets (N = 6). Black triangles: diabetic marmosets (N = 7). Diabetic marmosets showed a rapid decrease in blood glucose, indicating human insulin sensitivity. Values are the mean ± standard error. Note: some marmosets were not investigated due to their poor condition.

For a transplant model of human insulin-producing cells, an insulin tolerance test was performed to assess the sensitivity of marmosets to human insulin. Human insulin was subcutaneously injected into nonfasted animals at 3 U/kg. Blood samples were collected from the tail tip vein 0, 15, 30, 45, 60, 90, 120, 180 and 240 min after the glucose and insulin challenge. The blood glucose levels of the diabetic marmosets decreased in response to the administration of human insulin (Fig. 3).

### Glycated haemoglobin and glycoalbumin

**Figure 4:**
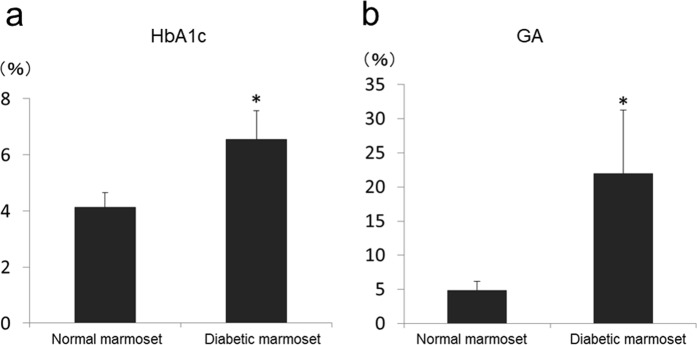
HbA1c and GA measurements in normal marmosets (N = 28) and diabetic marmosets (N = 19). HbA1c (**a**) and GA (**b**) values of diabetic marmosets were significantly higher than those of normal marmosets. *P < 0.05. Note: these tests were performed at several time points in each marmoset.

Serum glycated haemoglobin (HbA1c) and glycoalbumin (GA) levels were evaluated as glycaemic markers (Fig. 4). In normal marmosets, the serum HbA1c and GA levels were 4.13% ± 0.52% and 4.85% ± 1.34%, respectively. On the other hand, the diabetic marmoset serum HbA1c and GA levels were 6.54% ± 1.03% and 21.98% ± 9.29%, respectively, which are significantly higher than those of normal marmosets (P < 0.01).

### Blood chemistry

Before the experimental procedure, no abnormal blood chemistry findings were observed in any of the animals. After STZ injection, the total bilirubin level increased from 0.30 ± 0.13 mg/dl to 1.64 ± 1.08 mg/dl and the creatinine level increased from 0.11 ± 0.03 mg/dl to 0.38 ± 0.12 mg/dl. However, 1 or 2 months after the STZ injection, the total bilirubin level was decreased to 0.573 ± 0.19 mg/dl and the creatinine level was decreased to 0.28 ± 0.10 mg/dl. These results indicated that STZ induced temporary liver and renal damage.

### Histology

**Figure 5:**
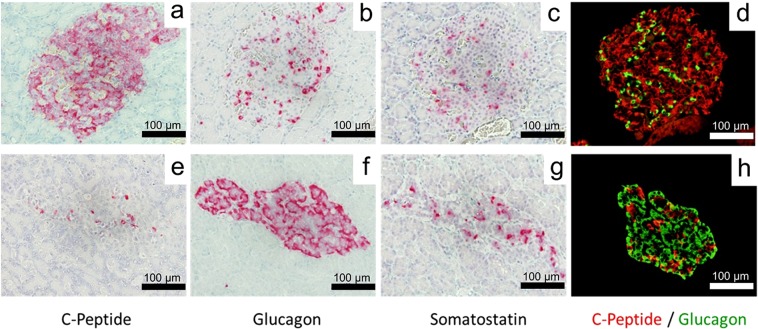
Histological and immunohistochemical analysis of the pancreas. Upper lane: normal (**a–d**). Lower lane: diabetic marmoset (**e–h**). C-peptide–positive cells are shown in red (**a,e**), glucagon-positive cells are shown in red (**b,f**) and somatostatin-positive cells are shown in red (**c,g**); C-peptide/glucagon double-immunofluorescent staining is also shown (**d,h**). Red: C-peptide-positive cells. Green: glucagon-positive cells.

Histological analysis was carried out both at the time of partial pancreatectomy and after the marmosets developed diabetes. Normal marmoset islets showed expression of C-peptide (Fig. 5a), glucagon (Fig. 5b) and somatostatin (Fig. 5c), and C-peptide–positive beta cells dominated. In normal marmosets, C-peptide/glucagon double-immunofluorescent staining (Fig. 5d) revealed that the proportion and distribution of C-peptide–positive beta cells and glucagon-positive alpha cells were similar to those of human pancreas. In the diabetic marmoset pancreas, the islet cells were damaged, or had even disappeared, with few C-peptide–positive cells (Fig. 5e,h) and higher numbers of glucagon-positive cells (Fig. 5f,h).

### Continuous glucose monitoring

**Figure 6:**
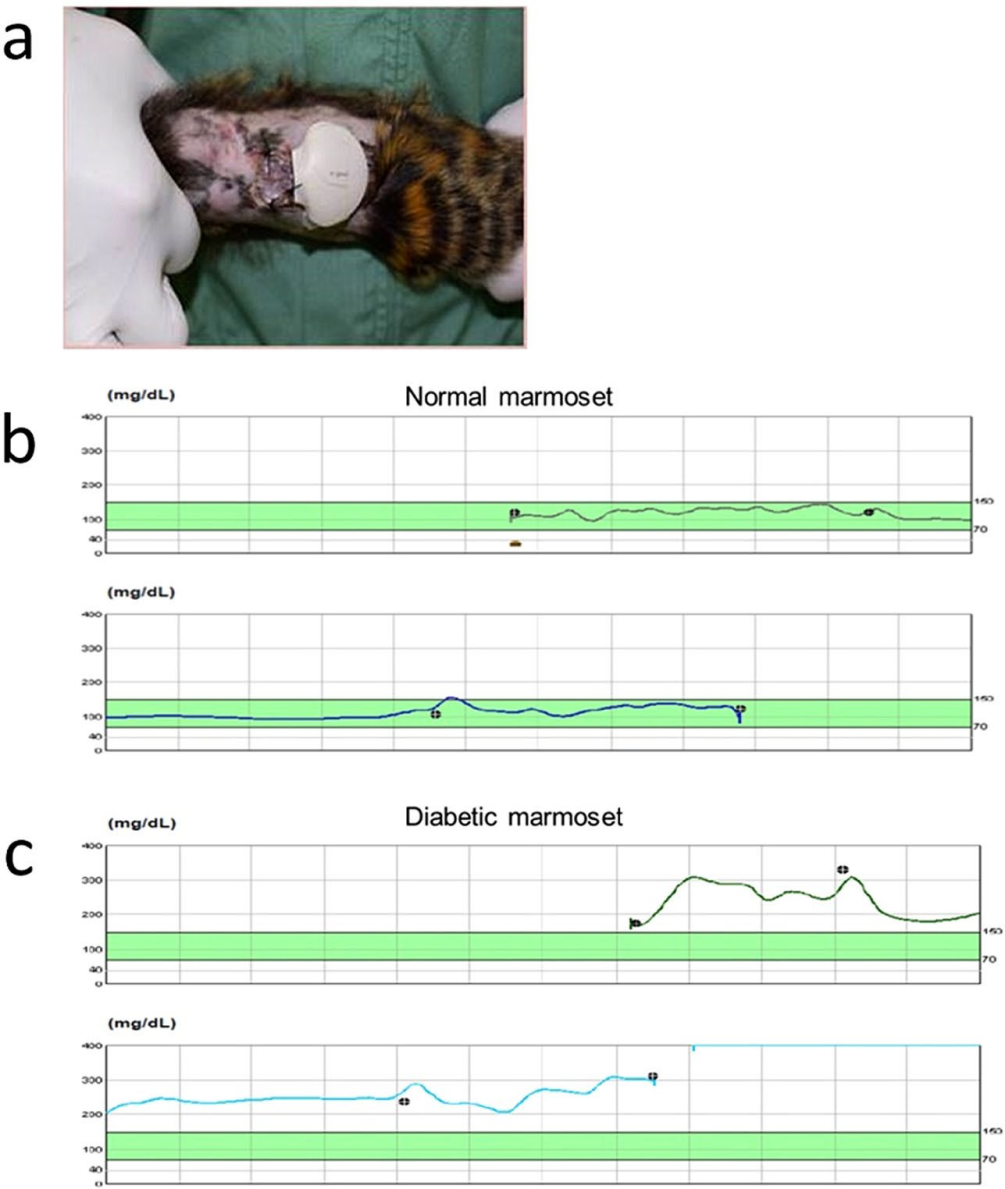
Continuous glucose monitoring. (**a**) Marmoset with an installed CGM device. (**b**) Blood glucose measurement in a normal marmoset showed almost the same range as in humans. (**c**) The diabetic marmoset showed persistently high blood glucose levels. After eating, the glucose levels showed greater fluctuation. The horizontal axis represents time (24 h in one row). The green bands indicate the normal human blood glucose range (70–150 mg/dl). Symbols show the blood glucose measurement time for calibration.

Continuous glucose monitoring (CGM) can provide a much more detailed and comprehensive picture of blood glucose control, even in sleeping animals. Because it is difficult to apply CGM to rodents, marmoset models can be useful. The sensor consists of a glucose oxidase-based platinum electrode that is introduced into the subcutaneous space using the needle stylet provided with the Medtronic ipro2 CGM system. Under inhalational anaesthesia, the marmoset’s fur was shaved from a 10-cm^2^ patch of skin on the back (Fig. 6a). After 24 hours, the CGM was removed and analysed. The normal marmoset blood glucose levels stayed in almost the same range as in healthy humans (Fig. 6b). After STZ administration and partial pancreatic resection, the diabetic marmoset showed persistently high blood glucose levels, particularly after eating (Fig. 6c).

## Discussion

Diabetes animal models are classified as either spontaneous models or artificial models according to pathogenesis. Diabetic animal models can be induced via various methods, such as via pancreatic resection, inheritable genetic modifications and chemical agents. Rodents, primates and other mammals have all contributed to diabetes research. In particular, spontaneous NHP diabetes models include those of macaques^14,15^, vervets^16^, baboons^17,18^ and mandrills^19^.

For preclinical research, particularly into cell transplantation therapy of diabetes, an NHP model is absolutely imperative. Because spontaneous type 1 diabetes mellitus occurs at an extremely low rate in NHPs^6^, NHP diabetic models are usually induced by pancreatectomy and/or STZ administration^12,20^. Although these models do not exhibit autoimmunity against beta cells, unlike type 1 diabetes mellitus patients, they are suitable for the assessment of graft cell function to improve glucose metabolism.

Total pancreatectomy reliably induces diabetes mellitus type 1, even in NHPs^21^. However, lack of insulin and glucagon can subsequently trigger brittle-type diabetes, and a lack of digestive hormones can provoke an absorption disorder, eventually permitting glycaemic control^22^.

STZ administration, as a nonsurgical approach, is a common, convenient and less invasive tool for inducing NHP diabetic models^12^. However, adjustment of the STZ diabetogenic dose is difficult. Pitkin *et al*. and Litwak *et al*. reported that low-dose STZ (20–50 mg/kg body weight) is not sufficient or reliable enough to consistently induce complete diabetes mellitus in cynomolgus monkeys^23,24^. On the other hand, Shibata *et al*.^25^ and Dufrane *et al*.^26^ reported that high-dose STZ (80–150 mg/kg body weight) could effectively induce diabetes in a rhesus monkey model and a cynomolgus monkey model, respectively, but was associated with more systemic side effects and serious complications. They also reported that high-dose STZ can induce hepatotoxicity and nephrotoxicity in diabetic NHPs.

The common marmoset is the smallest NHP model. In addition, its lifespan is relatively short compared with that of other NHPs. Nonetheless, marmosets’ pancreatic islet microarchitecture and glucose transporter expression are similar to those of humans^27^. However, STZ sensitivity varies according to species, with STZ shown to be less effective for the destruction of beta cells in marmosets^13^. This may be one reason why some of the marmosets did not become hyperglycaemic even after pancreatectomy and multiple STZ injections in this study.

In the present study, partial pancreatectomy (resection of approximately 70% of pancreatic tissue) combined with multiple STZ injections (100–160 mg/kg body weight) successfully achieved hyperglycaemia without significant adverse effects. As described above, Shibata *et al*.^25^ reported that high-dose STZ (80–150 mg/kg body weight) can trigger systemic side effects. There may be several explanations for the discrepancy between our results and those of the above studies. First, partial pancreatectomy reduced beta cell mass, decreasing the total STZ dose required for inducing diabetes. Second, we provided intensive care to the marmosets after the partial pancreatectomy and STZ administration, and we administered the next STZ injection after confirming that the animals had recovered from the damage of the prior treatment. Third, we used clinical-grade STZ, which has high purity.

Notable aspects of our study include (1) HbA1c and GA levels were significantly increased in diabetic marmosets compared with normal marmosets; (2) diabetic marmosets had significantly impaired glucose tolerance; (3) all marmosets showed high sensitivity to human insulin; and (4) blood glucose level variations, evaluated in real-time using CGM, revealed that our diabetic marmosets had similar blood glucose values to those of diabetic patients. Furthermore, as shown in Fig. 6, the blood glucose levels of diabetic marmosets fluctuate greatly, even within a single day. In addition, hyperglycaemic marmosets that were given insulin to sustain their lives sometimes became hypoglycaemic (Fig. 1). This mirrors the characteristic blood glucose instability of type 1 diabetes patients. After transplantation of insulin-producing cells (e.g., islet transplantation or stem cell-derived β-cell transplantation), we expect that this blood glucose instability will be resolved. CGM will be useful for the necessary evaluation because this test can adequately detect daily fluctuations.

On the other hand, it was difficult to maintain the condition of the diabetic marmosets for a long time, even though they were administered insulin injections on a daily basis. They exhibited signs of a slow deterioration with high aspartate aminotransferase and alanine aminotransferase) values (data not shown) indicating hepatic steatosis development. Nonetheless, their renal function was normal, even after more than 12 months. Thus, our diabetic marmoset model may not be suitable for long-term (>1 year) safety assessment if the animal remains diabetic. However, if blood glucose levels are normalized by transplantation of insulin-producing cells, there is a high probability that the animals will be maintained for a long time, as in other animal models. Of course, the model may be feasible for the evaluation of short- and medium-term functional effects after islet cell transplantation.

One limitation of this study is the lack of verification that this marmoset model can actually be used for cell transplantation. This study summarizes the development and evaluation of a marmoset diabetes model, and transplant experiments are planned as the next stage. However, as a preliminary study (data not shown), we transplanted insulinoma cell lines under the kidney capsule with the use of immunosuppressants and performed intraperitoneal transplantation of encapsulated porcine pancreatic islets. The transplants were safely concluded. Thus, the marmoset model is highly likely to be useful as a transplant model, but further research is required.

Another question concerns pancreatic regeneration after the pancreatectomy in the diabetic marmosets. This possibility has not been examined in this study. However, marmosets that developed hyperglycaemia remained in that state for more than a few months, until their general condition worsened due to diabetes. At least during this observation period, no large-scale regeneration was considered to have occurred. Further study is necessary in this regard as well.

In conclusion, we successfully induced diabetes in the common marmoset via a combination of partial pancreatectomy and STZ administration. Our marmoset model should be useful for diabetes research, particularly of cell therapies for diabetes.

## Methods

### Animals

This study was performed in strict accordance with the Regulation for Animal Experiments of the Central Institute for Experimental Animals (CIEA) (https://www.ciea.or.jp/en/3r/pdf/ae_kitei_bis_en.pdf) based on the Guidelines for Proper Conduct of Animal Experiments (Science Council of Japan, 2006). The animal experiment protocol was approved by the CIEA Institutional Animal Care and Use Committee (approval no. 14002A and 16036A). Blood tests were performed on all animals, and those with no abnormalities in body weight and appearance, blood counts or blood biochemical tests (e.g., liver function, kidney function, lipids, blood albumin and electrolytes) were used in this study. Eleven common marmosets (4–5 years old, 330–410 g, 4 females and 7 males) obtained from CLEA Japan (Tokyo, Japan) were used in this study. The untreated marmosets (i.e., before pre-treatment) were used as a healthy control group (normal marmoset).

### Partial pancreatectomy

**Figure 7:**
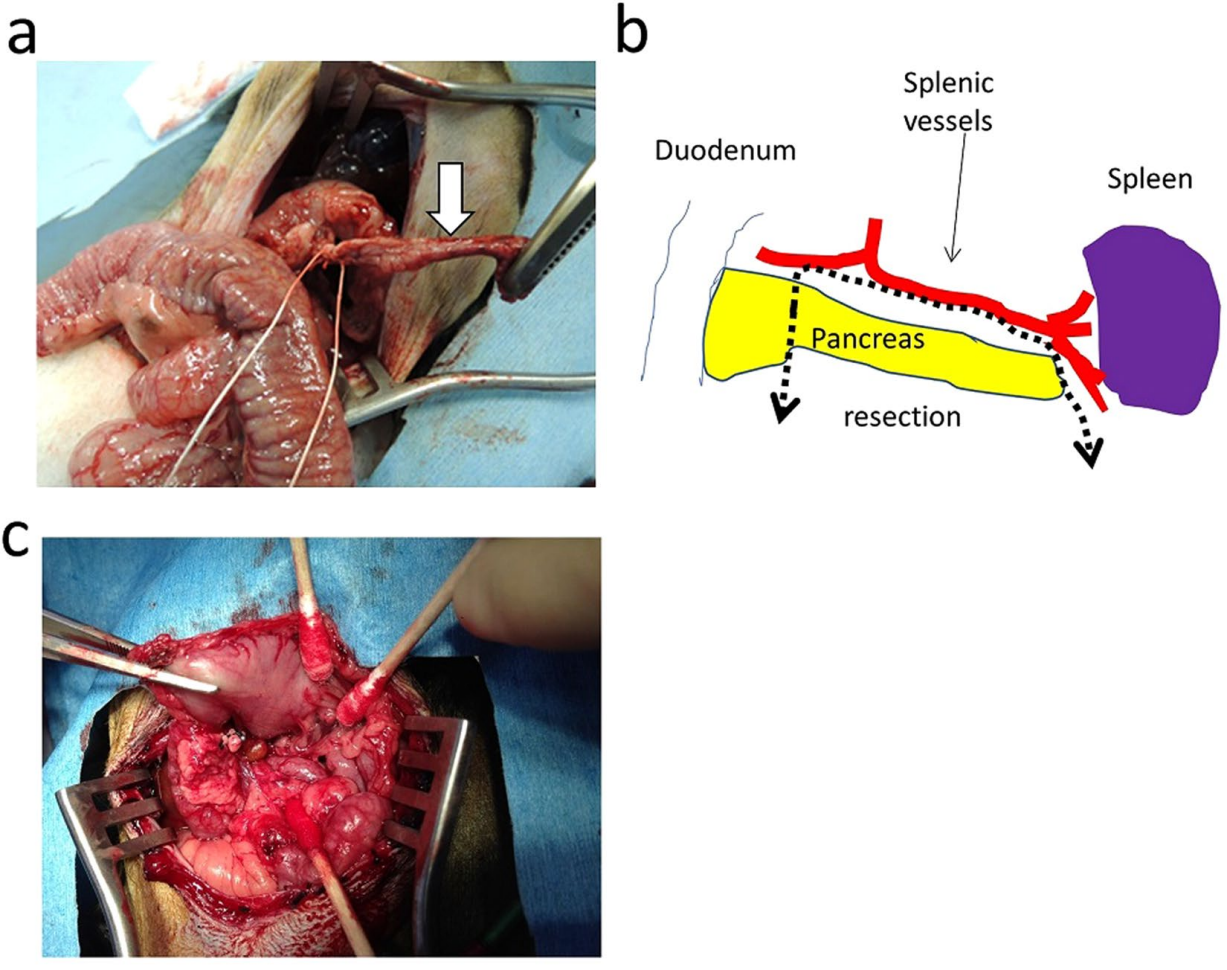
Partial pancreatectomy. (**a**) Ligated pancreas body and tail (white arrow) before resection. (**b**) Schematic of the partial pancreatectomy. Dotted line shows the resection line of the pancreas. (**c**) After the partial pancreatectomy.

Partial pancreatectomy was performed to induce diabetes. Marmosets were pre-anesthetized intramuscularly using ketamine (15 mg/kg), midazolam (0.15 mg/kg) and butorphanol (0.03 mg/kg). After intubation and induction of general anaesthesia with isoflurane, a partial pancreatectomy was performed; the animals were monitored using electrocardiography, a pulse oximeter and a thermometer. The surgery was performed based on a previous report^28^ on rhesus monkeys, with our modification. After midline laparotomy, the stomach and greater omentum were retracted towards the subphrenic space and the transverse colon was pulled to the tail side to expose the surface of the pancreas. The peritonea over the superior and inferior borders of the pancreas were then divided. Starting laterally adjacent to the spleen, the tail of the pancreas was detached from its posterior attachments. The upper and lower margins of the pancreatic tail were also freed of their mesenteric attachments. The tail of the pancreas was isolated, and the spleen with its vessels was carefully protected. Then, the neck and body of the pancreas were isolated. Multiple small vessels were obliterated by electrocoagulation and divided. The pancreas was ligated between the head and neck at the level of the superior mesenteric vein, after which the neck, body and tail of the pancreas were removed. Approximately 70% of the pancreas was removed by this procedure (Fig. 7a–c). As post-operative analgesia and infection control, ketoprofen (1.2 mg/kg) and ampicillin (15 mg/kg) were intramuscularly administered to the animals once daily for 3 consecutive days.

### Streptozotocin injection

STZ is a naturally occurring chemical that is particularly toxic to the insulin-producing beta cells of the mammalian pancreas. Zanosar (Teva Pharmaceuticals Industries, Petah Tikva, Israel) is a clinical-grade form of STZ with greater purity and less variability that is associated with fewer adverse effects than other preparations of STZ used for diabetes induction. More than 2 weeks after partial pancreatectomy, the marmosets were fasted overnight. STZ, dissolved in saline, was administered by intravenous injection at a dose of 160 mg/kg body weight for more than 30 seconds. Nonfasting blood glucose levels were measured twice a week after STZ injection. When the nonfasting blood glucose level consistently exceeded 200 mg/dl and the spot urine test became glucose-positive, the marmoset was considered to be diabetic. When the marmoset did not become diabetic after a single STZ administration, additional STZ was injected at a dose of 100–160 mg/kg body weight. After the successful induction of diabetes, the marmosets were treated with human insulin to avoid metabolic dysfunction and maintain a good general condition.

### Metabolic studies

IVGTTs and OGTTs were performed after overnight fasting. For the IVGTT, 0.5 g/kg body weight glucose solution was intravenously injected. Blood samples for measurement of insulin levels were collected at 0, 15 and 60 min, whereas blood glucose levels were estimated at 0, 5, 15, 30, 45, 60 and 90 min. For the OGTT, 2 g/kg body weight glucose solution was orally administered. Blood samples for insulin levels were collected at 0, 30 and 120 min, whereas blood glucose levels were estimated at 0, 15, 30, 60, 90, 120 and 180 min. All serum samples were sent for analysis to a trusted external company (Oriental Yeast Co., Ltd., Tokyo, Japan), and serum insulin was measured by a radioimmunoassay kit (PI-12K Porcine Insulin RIA-kit; Millipore, St. Charles, MO). Serum HbA1c and GA levels were measured preoperatively and several times in the 1 to 4 months after the animals developed diabetes.

### Insulin tolerance test

To evaluate insulin sensitivity, 1 U/kg body weight of insulin (Humulin R, Eli Lilly Japan K.K., Kobe, Japan) was subcutaneously injected into nonfasted animals. Blood samples were collected from the tail tip vein 0, 15, 30, 45, 60, 90, 120, 180 and 240 min after insulin challenge.

### Blood chemistry and urinalysis

For biochemical assessments, including hepatic and renal function tests, serum was collected before the procedure and twice a week after partial pancreatectomy and STZ administration. Biochemical parameters were measured using an automatic analyser (DRI-CHEM 7000 V; Fujifilm, Tokyo, Japan). Spot urine samples were obtained twice a week and measured by a urine dipstick test.

### Continuous glucose monitoring

Under inhalational anaesthesia, a CGM system sensor (Medtronic ipro2; Medtronic Inc., Tokyo, Japan) was placed under the skin of the marmosets. This CGM system has been approved in Japan for the evaluation of daily glucose profiles in human patients with diabetes. The device estimates blood glucose values every 5 min over 3 days by measuring interstitial glucose concentrations; the calibration readings are integrated with the raw CGM data at the time of data upload using the web software.

### Histology

Normal pancreatic tissue was obtained at the time of partial pancreatectomy. Diabetic pancreatic tissue was obtained after animals were diagnosed with diabetes. Pancreatic tissue was fixed in 10% formalin neutral buffer solution, embedded in paraffin and cut into 3-µm-thick sections. Pancreas tissues were stained for C-peptide, glucagon and somatostatin via fluorescent staining and alkaline phosphatase staining. For the fluorescent staining, the tissue was incubated overnight at 4 °C with the primary antibody: C-peptide antibody (1:250, LS-C45862, Lifespan Biosciences, Seattle, WA), glucagon antibody (1:200, #8233, Cell Signaling Technology, Danvers, MA) or somatostatin antibody (rabbit polyclonal IgG, 1:250, LS-C172192, Lifespan Biosciences). The next day, the tissue was incubated with the appropriate secondary antibodies: Alexa Fluor® 594 (red; 1:300, Life Technologies, Carlsbad, CA) and Alexa Fluor® 488 (green; 1:300 Life Technologies); a VECTASTAIN ABC-AP Kit (Vector Laboratories, Burlingame, CA) was used for the alkaline phosphatase staining.

### Statistical analysis

Student’s unpaired, two-tailed *t*-test was used to compare the two groups.

## Supplementary information

Supplementary information

## Data Availability

The datasets generated during and/or analysed during the current study are available from the corresponding author on reasonable request.
