# Supplementary material for: Establishment of a diabetes mellitus type 1 model in the common marmoset
Source: Sci Rep. 2019 Oct 10;9:14546. doi: 10.1038/s41598-019-51199-5 (PMC6787219; doi:10.1038/s41598-019-51199-5)
Supplement: Supplementary file 1 — Supplementary information [file 41598_2019_51199_MOESM1_ESM.docx]

Supplementary information

**Establishment of a diabetes mellitus type 1 model in the common marmoset**

Wenji Yuan^1^, Satsuki Fukuda^2^, Takashi Inoue^3^, Hitoshi Okochi^2^, Erika Sasaki^3^, and Masayuki Shimoda^1,*^

^1^Department of Pancreatic Islet Cell Transplantation, National Center for Global Health and Medicine, 1-21-1 Toyama, Shinjuku-ku, Tokyo 162-8655, Japan

^2^Department of Regenerative Medicine, National Center for Global Health and Medicine, 1-21-1 Toyama, Shinjuku-ku, Tokyo 162-8655, Japan

^3^Department of Marmoset Biology and Medicine, Central Institute for Experimental Animals, 3 Chome-25-12 Tonomachi, Kawasaki-ku, Kawasaki-shi, Kanagawa-ken 210-0821, Japan

**Corresponding author:** Masayuki Shimoda

Islet Cell Transplantation Project, Diabetes Research Center, Research Institute of National Center for Global Health and Medicine, 1-21-1 Toyama, Shinjuku-ku, Tokyo 162-8655, Japan

**Tel:** +81-3-3202-7181

**E-mail:** mshimoda@hosp.ncgm.go.jp

Supplemental figure S1


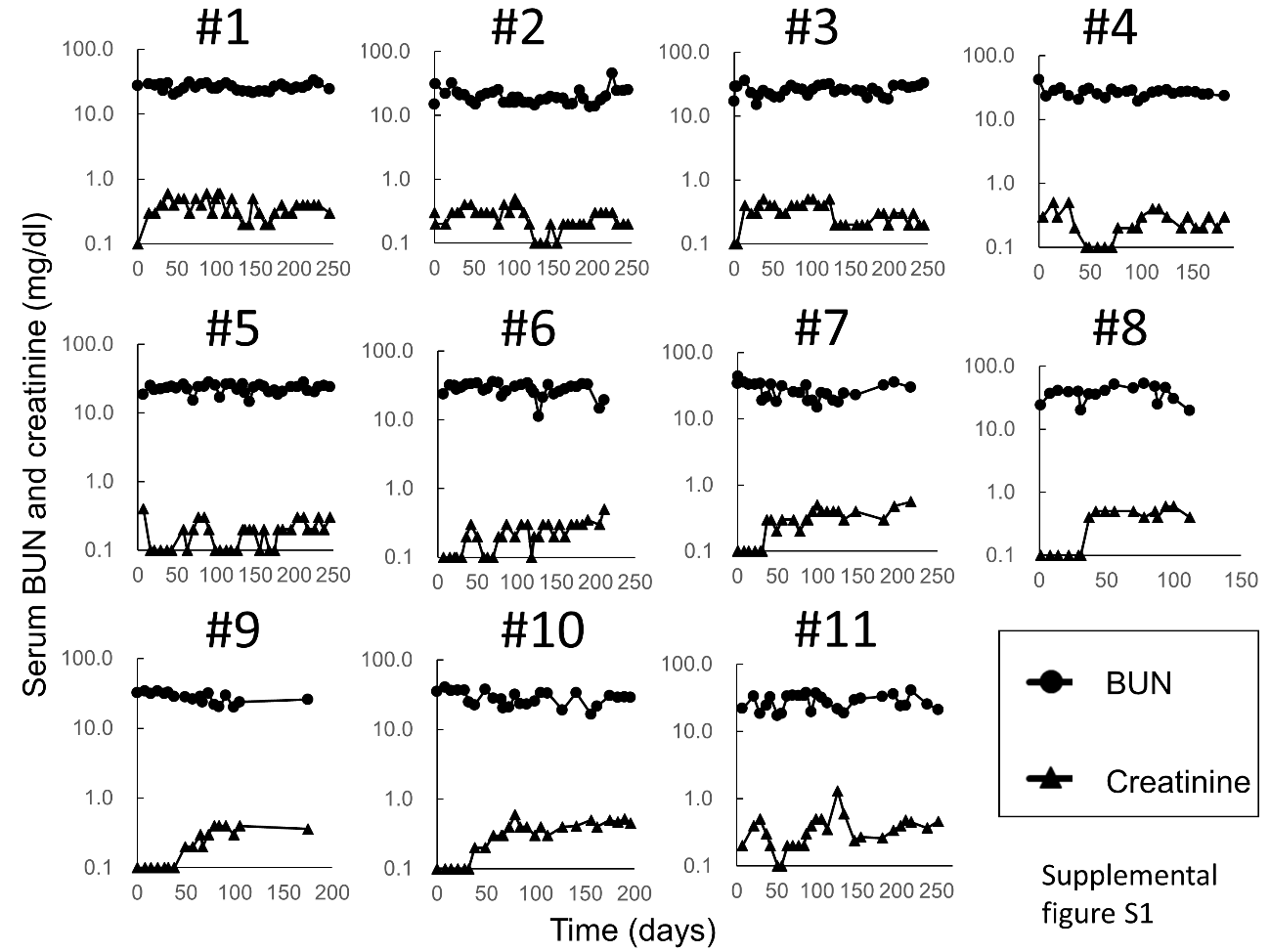


Supplemental figure S2


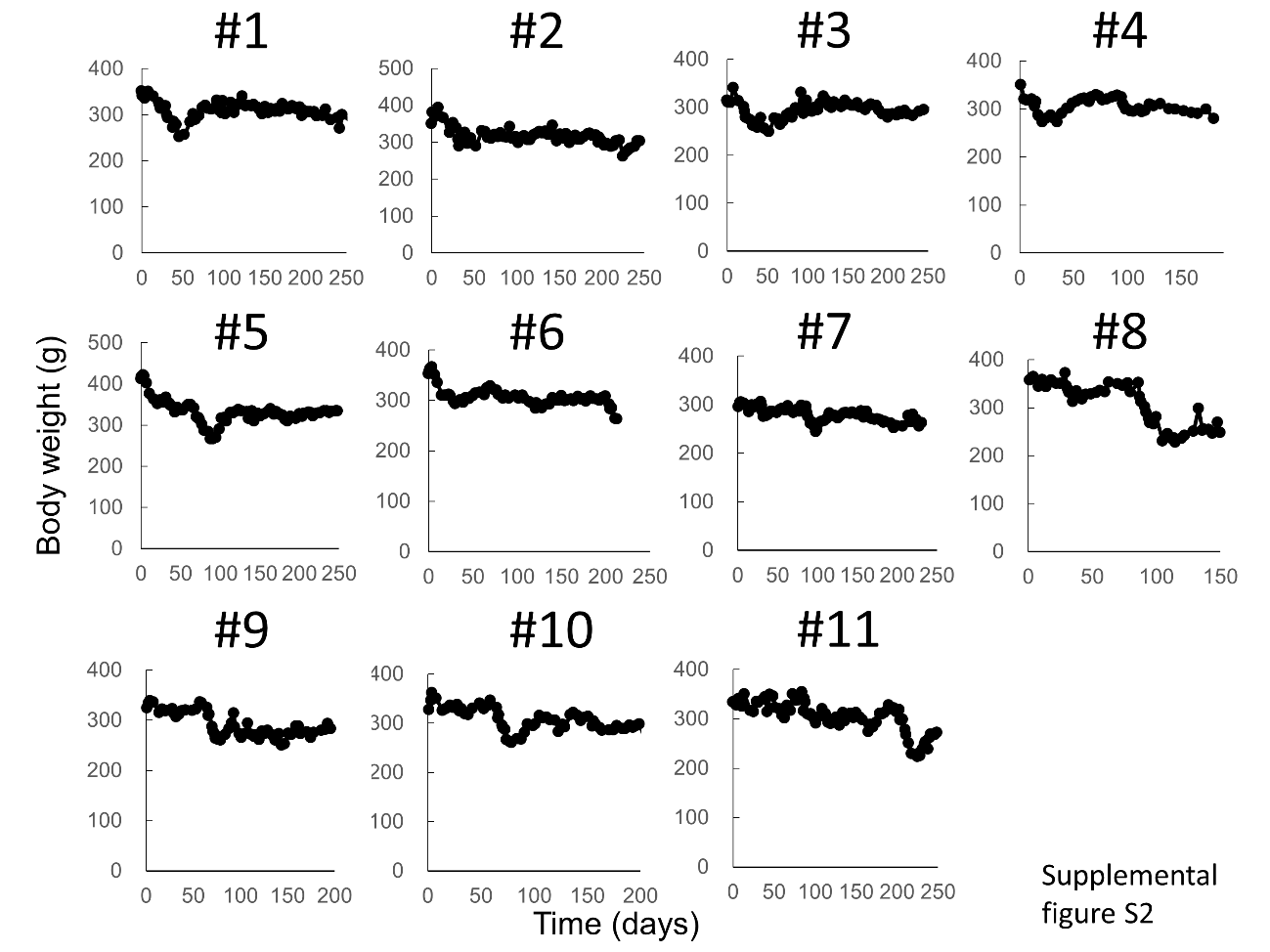


**Supplemental figure legends**

Supplemental figure S1. Renal function changes of individual marmosets after partial pancreatectomy and administration of STZ. The horizontal axis represents the number of days after the partial pancreatectomy. The vertical axis represents blood urea nitrogen (BUN) (mg/dl) and blood creatinine (mg/dl). Black circles: BUN. Black triangles: creatinine.

Supplemental figure S2. Body weight changes of individual marmosets after partial pancreatectomy and administration of STZ. The horizontal axis represents the number of days after the partial pancreatectomy. The vertical axis represents the body weight (g).
